# Supplementary material for: Hypothermia evoked by stimulation of medial preoptic nucleus protects the brain in a mouse model of ischaemia
Source: Nat Commun. 2022 Nov 12;13:6890. doi: 10.1038/s41467-022-34735-2 (PMC9653397; doi:10.1038/s41467-022-34735-2)
Supplement: Supplementary file 1 — Supplementary Information [file 41467_2022_34735_MOESM1_ESM.pdf]

Supplementary Information

Hypothermia evoked by stimulation of medial preoptic nucleus protects the brain in a mouse model of ischaemia

Shuai Zhang[1,#] Xinpei Zhang[1,#], Haolin Zhong[1], Xuanyi Li[1], Yujie Wu[1], Jun Ju[1], Bo Liu[1], Zhenyu Zhang[1], Hai Yan[1], Yizheng Wang[2], Kun Song[1,*], Sheng-Tao Hou[1,*]

[1] Brain Research Centre, Department of Biology, School of Life Sciences, Southern University of Science and Technology, 1088 Xueyuan Blvd, Nanshan District, Shenzhen, Guangdong Province, 518055, P. R. China,
[2] Huashan Hospital, Fudan University, Shanghai, P. R. China.

[#] These authors contributed equally: Shuai Zhang, Xinpei Zhang

* Corresponding authors:
Associate Prof. Kun Song, email: songk@sustech.edu.cn, and
Prof. Sheng-Tao Hou, email: hou.st@sustech.edu.cn (Lead Contact).

Supplementary Information List:

1. Supplementary Figures (p.2-13)
2. Supplementary Table 1 (p.14)

1. Supplementary Figures

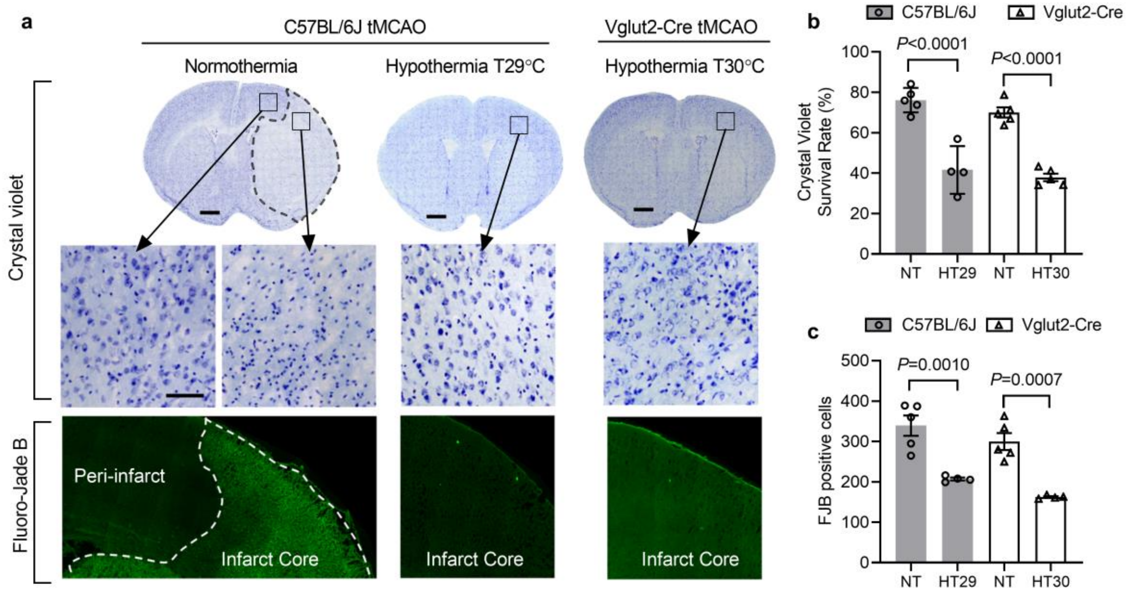

Supplementary Fig. 1 | Histochemical staining of coronal brain sections. **a** Coronal brain sections were cut at 16 µm thickness and subjected to staining with Crystal violet and FJB. Dotted-lined areas indicated ischaemic infarct core (scale bar = 1 mm). Areas indicated by small black boxes were shown in higher magnifications as arrows indicated (scale bar = 20 µm). The percentage of healthy cells under crystal violet was counted and plotted in **b** and FJB positive degenerating cells were plotted in **c**. 1ANOVA was performed with Tukey's *post hoc* analysis (**b**, $F_{(3, 15)} = 35.68$, $P < 0.0001$; **c**, $F_{(3, 14)} = 19.07$, $P < 0.0001$. The P values of specific comparison between groups were indicated in the graph of **b** and **c**). All data were mean ± s.e.m ($n = 5$ mice). Source data are provided as a Source Data file.

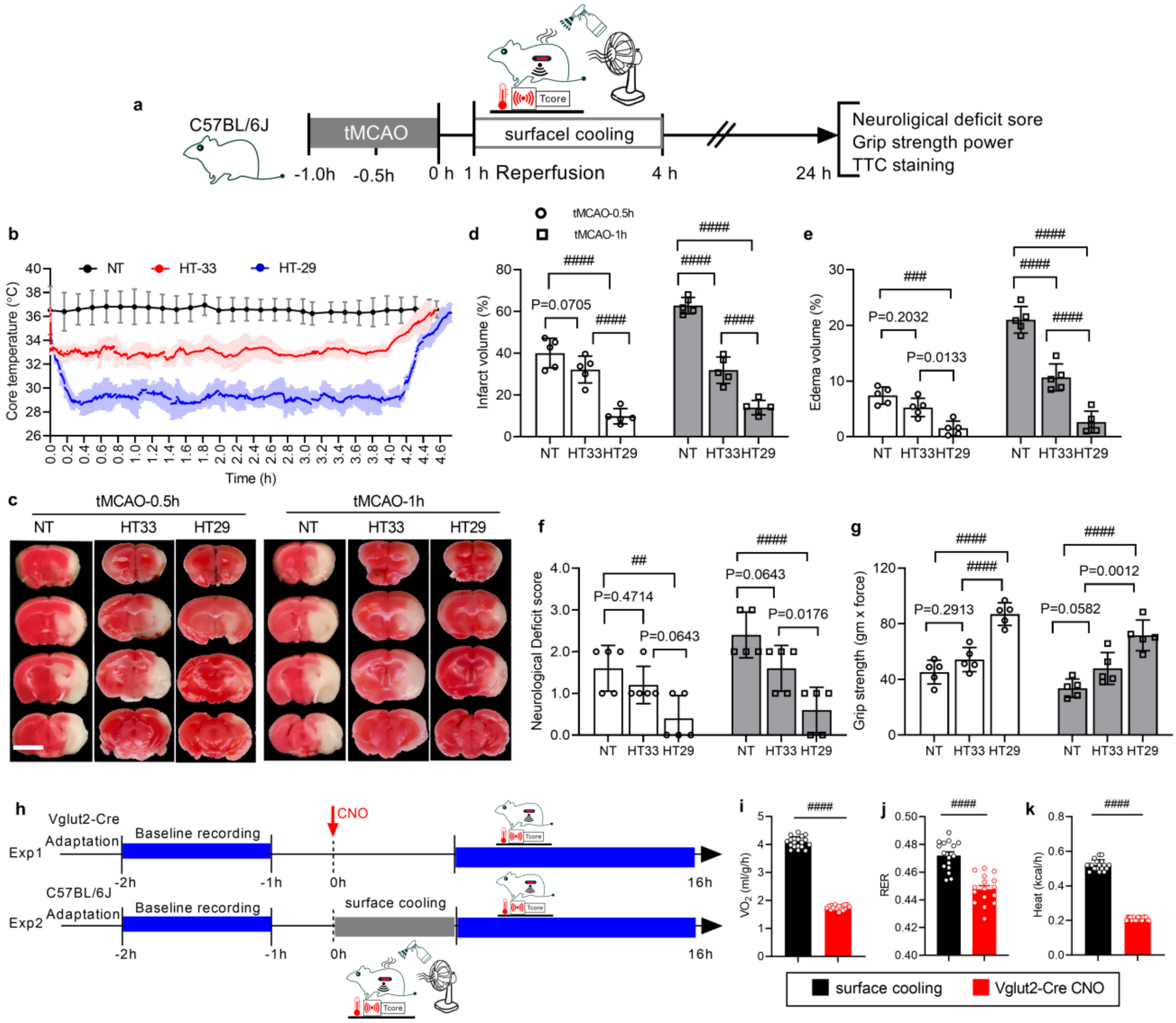

Supplementary Fig. 2 | Surface cooling-triggered hypothermia protected ischaemic mouse brain. **a** Schematic of experimental design. C57BL/6J male mice at 6-7 weeks old with a bodyweight of 23 g were subjected to tMCAO for 0.5 h or 1 h. After 1 h reperfusion, mice were induced hypothermia for a specified time and reperfusion for 24 h. Mice behavioural tests were performed as indicated before being killed for brain section staining with TTC (**c**). **b** Recordings of T_{core} were plotted against the reperfusion time for 4 h to show stable T_{core} at 33 °C (red-coloured line) and 29 °C (blue-coloured line). **c** Brain coronal sections at 2 mm thickness were stained with TTC to show viable tissue (red colour) and dead tissue (white colour). Scale bar = 5 mm. Measurements of tMCAO brain infarction (**d**), oedema volumes (**e**), neurological deficits scores (**f**), and forepaw grip strength (**g**). 2ANOVA with Tukey's *post hoc* test for significant groups, $^{\#\#\#\#}$ indicating $P < 0.0001$. **h** Schematic for metabolic rate measurements. The average levels of reduction in VO_2 (**i**), RER (**j**), and heat (**k**) were calculated. The average of all the time points were plotted. Unpaired two-tailed t test was performed with $^{\#\#\#\#}$ indicating $P < 0.0001$. All data were mean ± s.e.m ($n = 5$ mice). Source data are provided as a Source Data file.

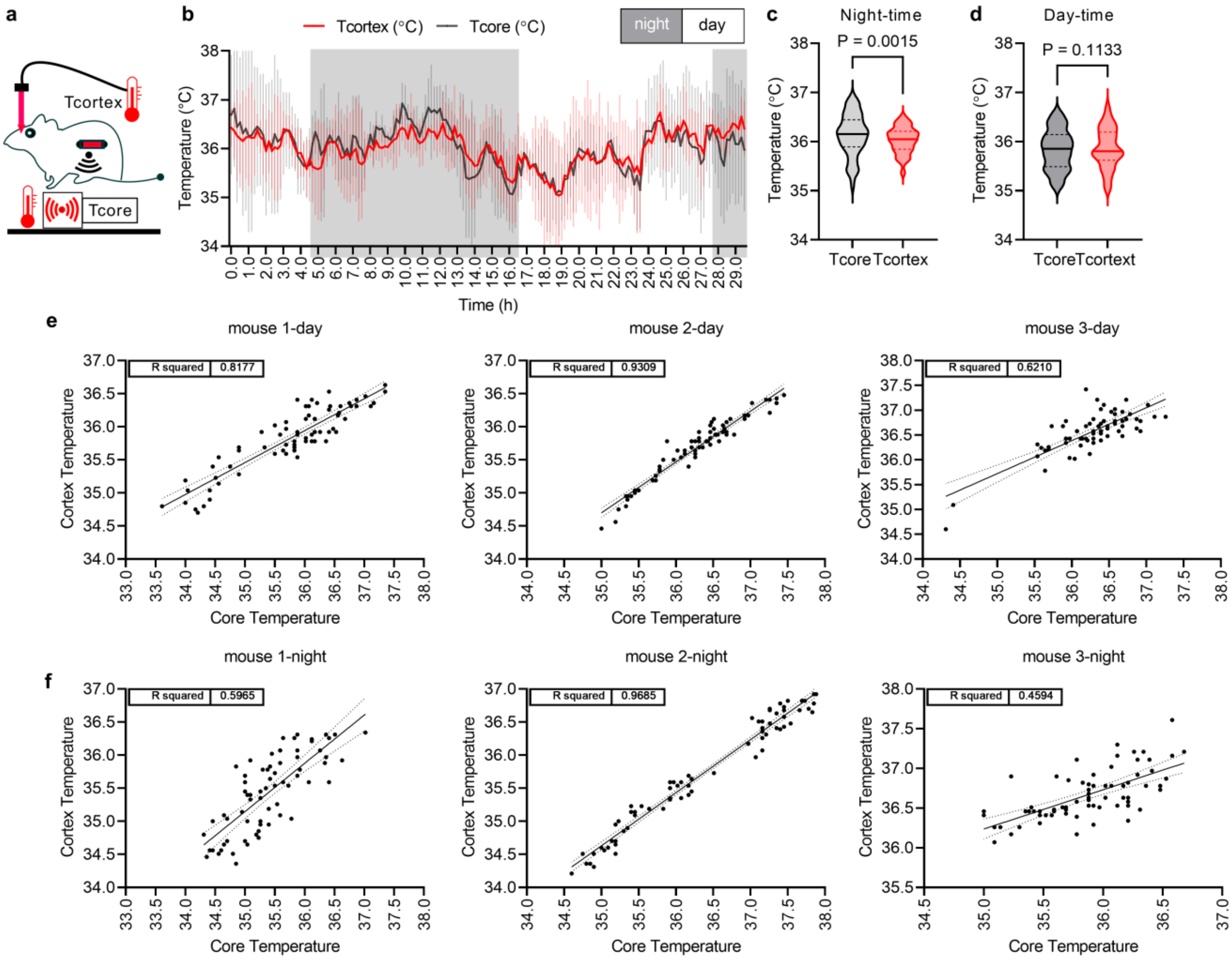

Supplementary Fig. 3 | Determination of the relationship between the brain and core body temperatures. a Schematic for recordings of T_{cortex} and T_{core} simultaneously in the free-moving mouse. **b** Plots of T_{cortex} and T_{core} recordings over 29 h. The grey-coloured area indicated night-time, and the white area indicated day-time. The average values of the night-time and day-time T_{cortex} and T_{core} were calculated and plotted in **c** and **d,** respectively. The solid line indicates the median and the dotted lines for quartiles. Paired two-tailed t test was performed for the night-time $^{\#\#\#}P_{(\mathrm{Tcore\ vs.\ Tcortex})} = 0.0015$ [t(66) = 3.305), and the day-time $P_{(\mathrm{Tcore\ vs.\ Tcortex})} = 0.1133$ [t(57) = 1.608); n.s.= not significant. Data were mean ± s.e.m ($n = 3$ mice). The Pearson's correlation coefficient (R^2) between T_{cortex} and T_{core} of each mouse was calculated and plotted in **e** and **f**. Source data are provided as a Source Data file.

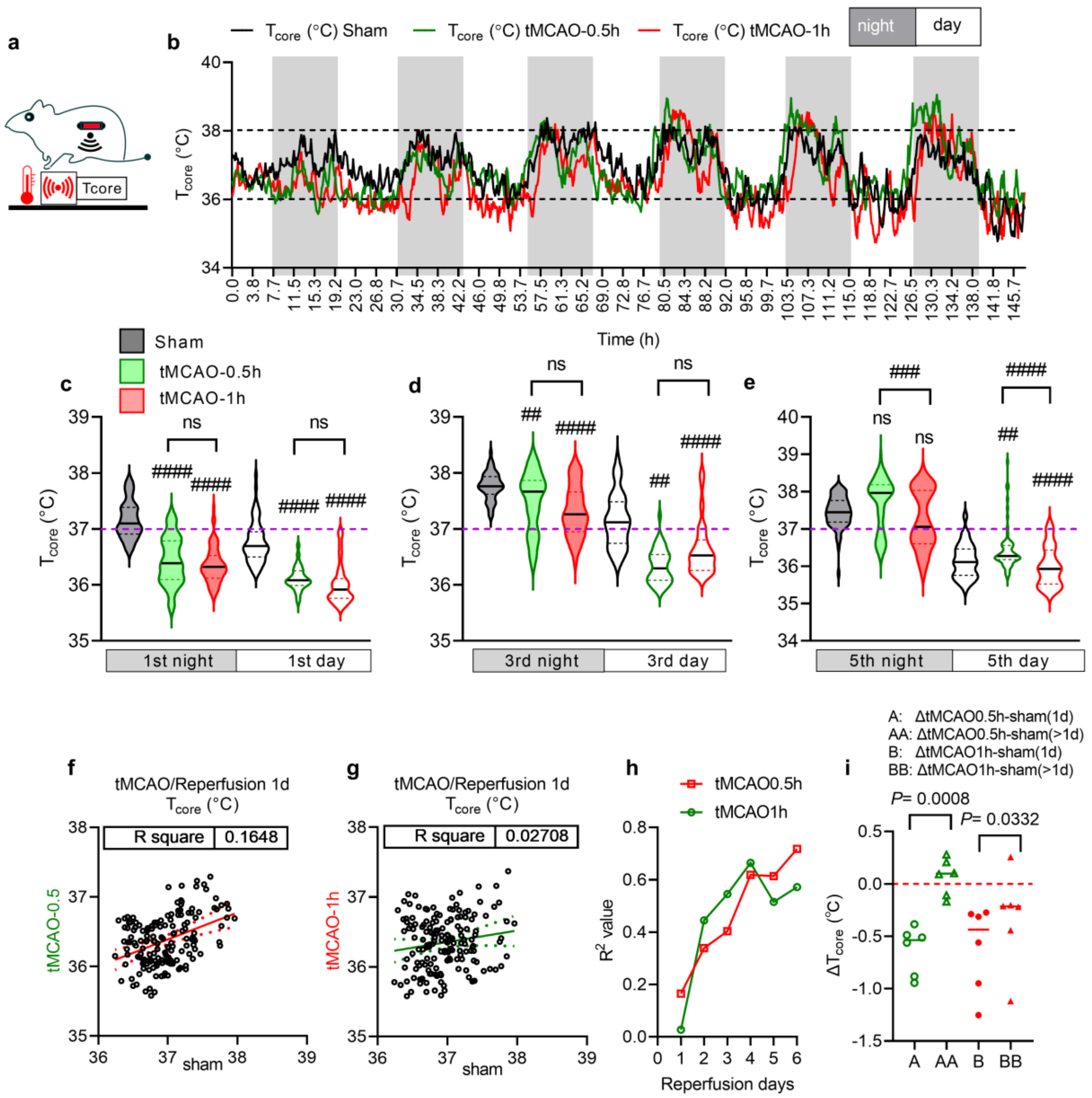

Supplementary Fig. 4 | Tacking T_{core} changes after cerebral ischaemia/reperfusion. **a** Schematic of T_{core} recording using an implanted temperature telemetric transmitter in a free-moving mouse. **b** Recordings of T_{core} were plotted against the 5 d reperfusion time. Grey-colored boxes indicated the night-time, and the white area in between indicated the day-time, showing a clear circadian rhythm for T_{core} 2 d after tMCAO. **c-e** Violin plots for the average night- and day-time T_{core} of the Sham (dark grey), tMCAO-0.5h (green), and tMCAO-1h (red) group of mice at 1 d (**c**), 3d (**d**), and 5 d (**e**) reperfusion to illustrate disruptions of T_{core} after tMCAO. The purple-coloured dash line in the plot indicated the mean T_{core} of the Sham mice. The solid line indicates the median and the dotted lines for quartiles. 1ANOVA with Tukey's *post hoc* test was used to identify statistical differences between Sham and the tMCAO groups. The specific P values can be found in the Supplementary Table 1. n.s.= not significant, $^{\#\#}P < 0.05$, $^{\#\#\#}P < 0.01$, and $^{\#\#\#\#}P < 0.001$. **f-h** Pearson's correlation of T_{core} between the Sham and two tMCAO groups during the 1 d reperfusion (**f,g**) and the upward trend of of R^2 values

during reperfusion (**h**). **i** The ΔT_{core} between the Sham and tMCAO groups at 1 d (indicated as A and B) and the average of the 2-5 d reperfusion period (indicated as AA and BB). Paired two-tailed t test was performed: $P_{(AA\ vs.\ A)} = 0.0008$ [t(5) = 6.178]; $P_{(BB\ vs.\ B)} = 0.0332$ [t(5) = 2.34]. All data were mean ± s.e.m (*n* = 5 mice). Source data are provided as a Source Data file.

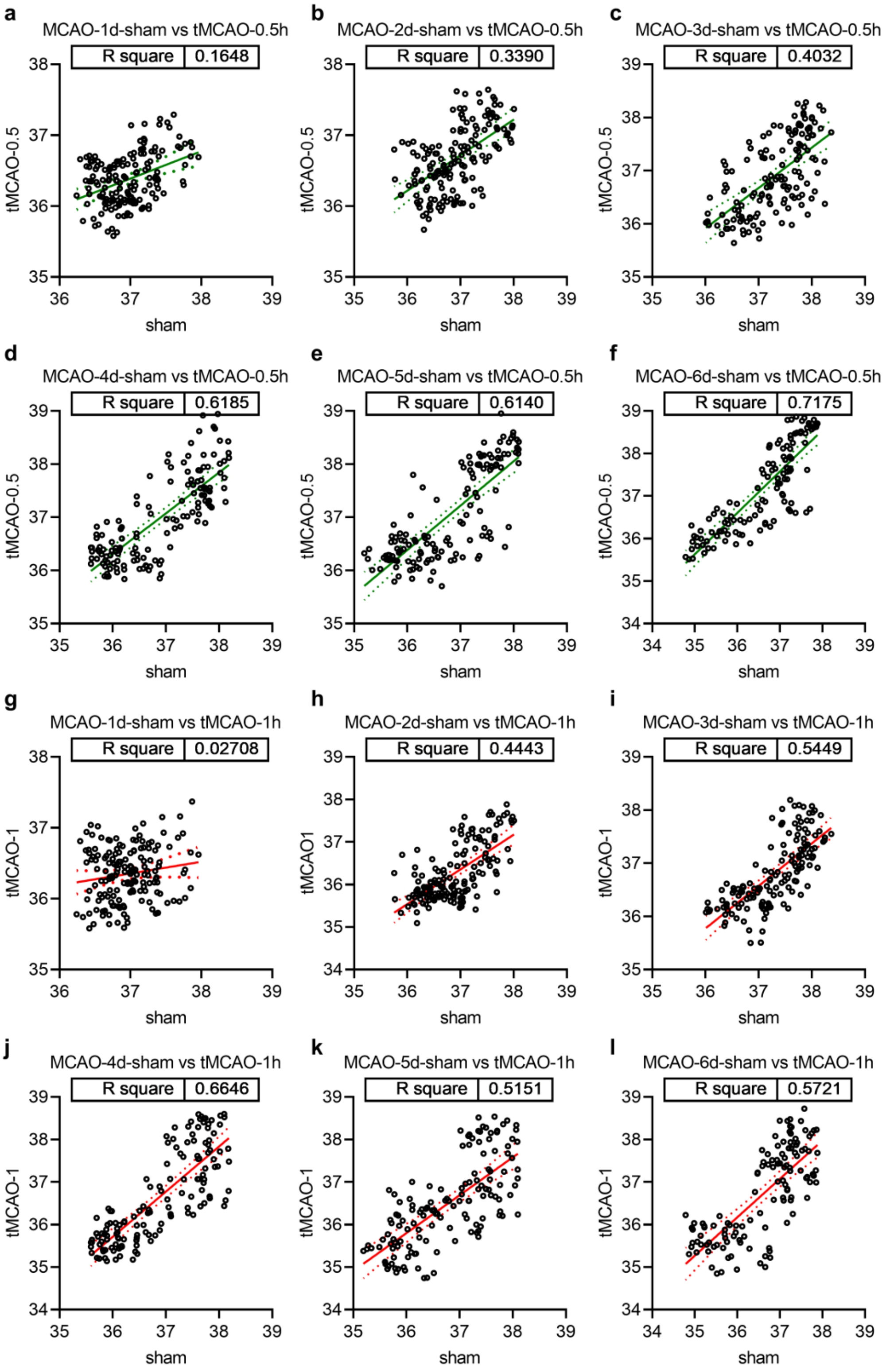

Supplementary Fig. 5 | Correlation of T_{core} changes between the tMCAO and sham groups during reperfusion. a – l Pearson's correlation analyses of T_{core} between the Sham and tMCAO-0.5h group and the Sham and tMCAO-1h group every day during reperfusion. The R^2 values were as indicated. All data were mean ± s.e.m (n = 5 mice). Source data are provided as a Source Data file.

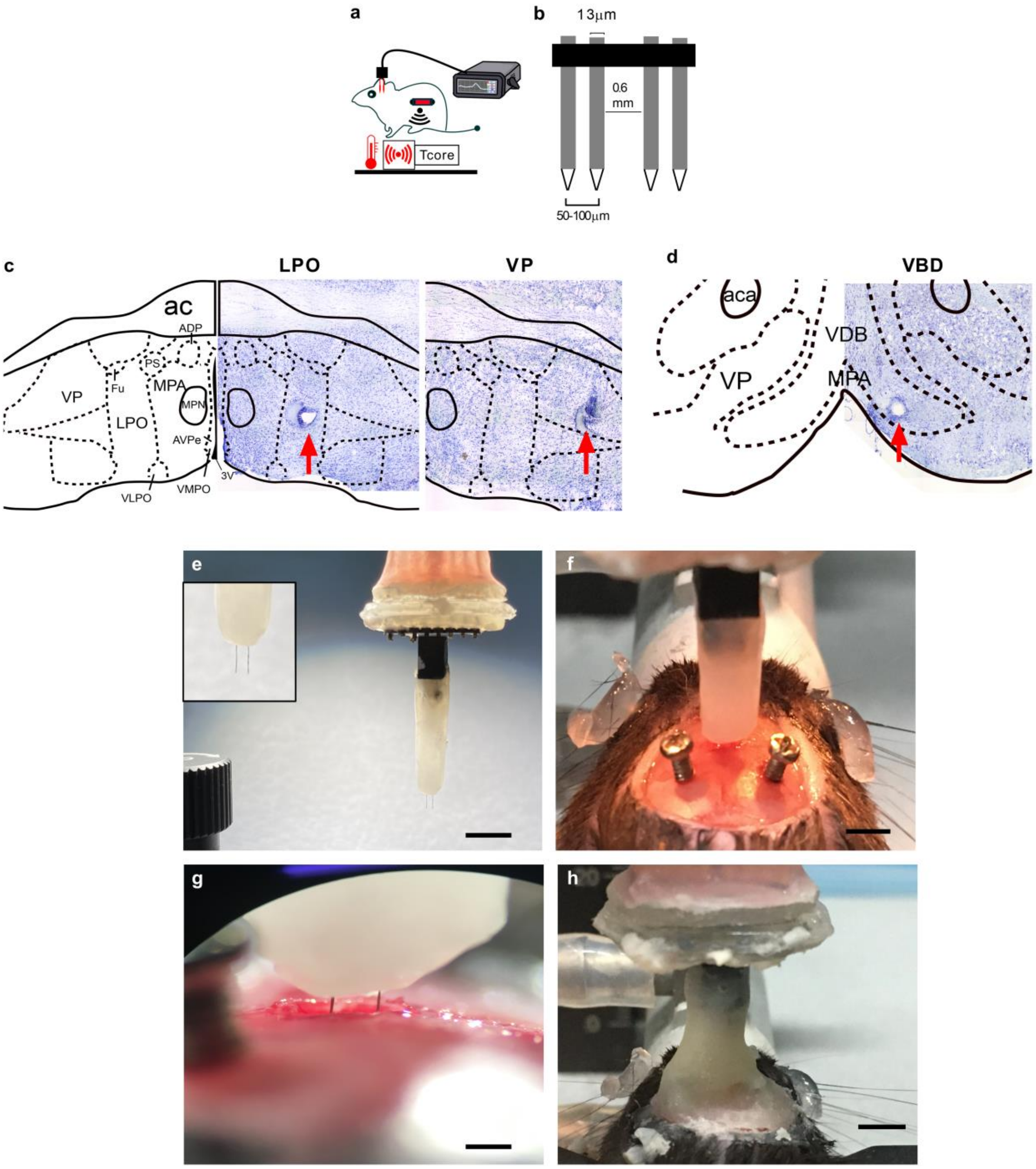

Supplementary Fig. 6 | Deep brain stimulation locations and surgical setup. a Schematic of electrical DBS setup. The mouse was implanted with a bipolar stimulating electrode and a telemetric transmitter to record T_{core}. A diagram of the bipolar electrode was shown in (**b**) (diagram not to scale). **c,d** The mouse brain section with crystal violet stating after electoral lesion to indicate the DBS electrode site in the LPO, VP and VBD. (red arrow indicate the electrode site). **e** The electrical stimulation electrodes construction and the tip of the dipole electrode (inset in **e**). **f, g** Photos depicting procedures of implanting electrodes into the brain. **h** Photos of the final stage of securing the electrodes with dental cement. Scale bars = 3 mm.

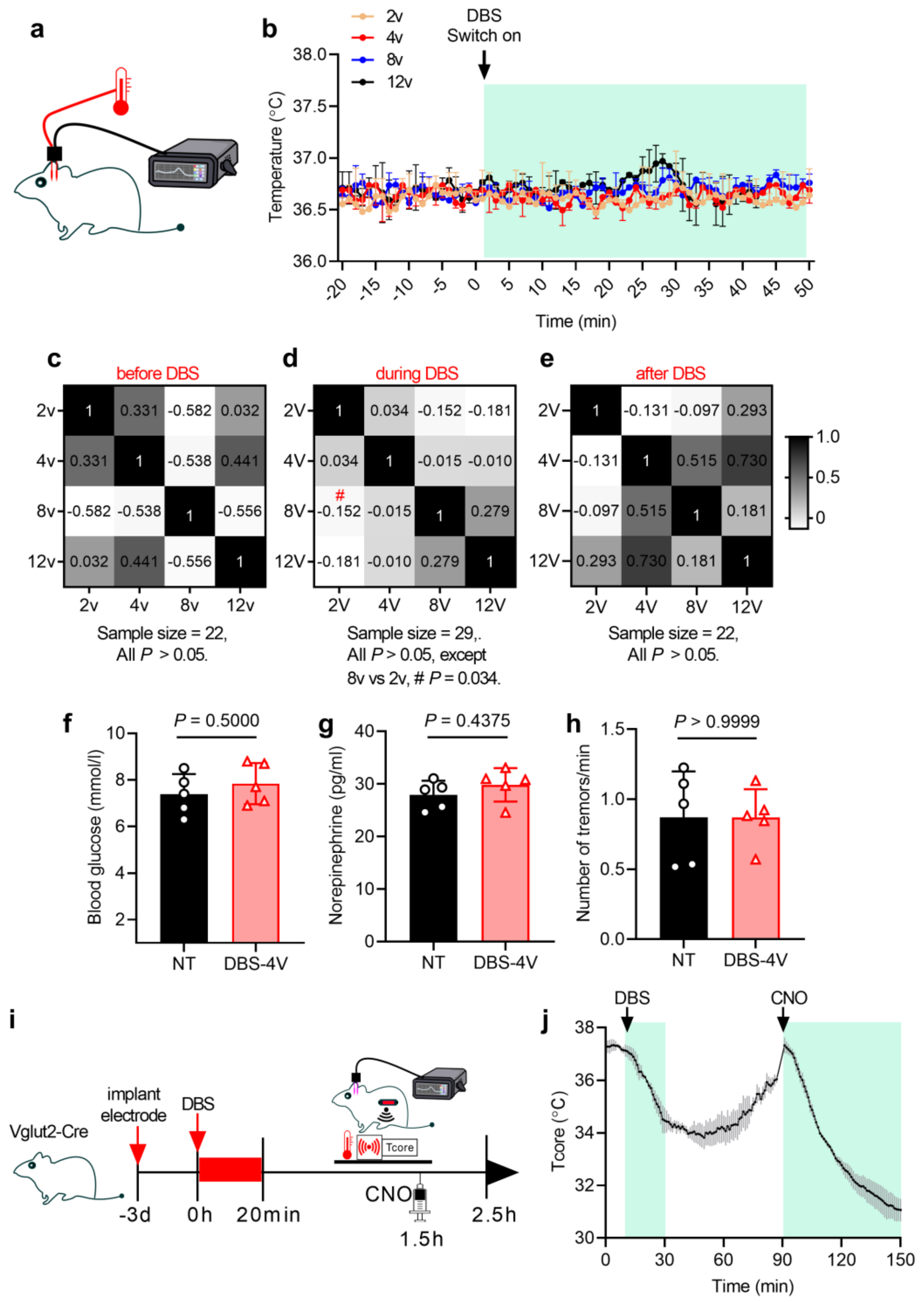

Supplementary Fig. 7 | Tracking DBS-induced temperature and physiological changes. a Schematic of electrical DBS and temperature probe setup. **b** Temperature traces during the 50 min period of electrical DBS with various voltages at 2 V, 4 V, 8 V, and 12 V. The arrow indicated the time of switching on DBS. The green-coloured area indicates the recording time window. **c-e** Heatmaps showing Spearman correlation coefficient matrix (two-tailed) of T_{core} before (**c**), during (**d**) and after (**e**) DBS at the voltage indicated. The r_s values were as indicated. n = 3 mice. The blood levels of

glucose (**f**) and norepinephrine (**g**) from DBS mice (DBS-4V) and normothermic mice (NT) were shown. **h** The number of shivering tremors was recorded and plotted. Nonparametric two-tailed Wilcoxon test. n = 5 mice. **i** Schematic of experimental design. Vglut2-Cre mice injected with Gq-DREADD-AAV for two weeks were subjected to DBS (30 min). After the T_{core} recovered to normal, CNO was injected i.p. to re-evoke hypothermia (**j**). All data were mean ± s.e.m (n = 5 mice). Source data are provided as a Source Data file.

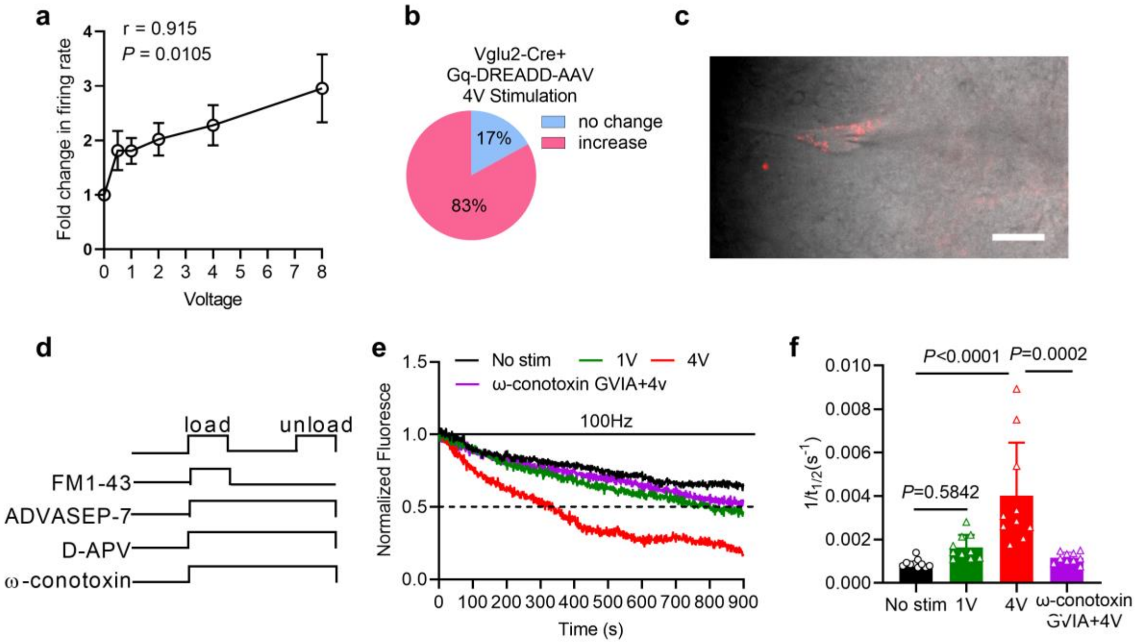

Supplementary Fig. 8 | Evidence showing DBS activation of WSNs in the medial preoptic nucleus. a Increased fold changes in firing rate of neurones from Vglut2-Cre-DREADD-AAV mice brain slice (two-tailed Pearson's correlation test). **b** The total numbers of recorded Vglut2-Cre-Gq-DREADD-AAV-mCherry positive cells which responded to 4 V HFS (red coloured) and the non-responders (blue coloured). **c** A photograph showing patch clamping of a mCherry positive neurone (orange-colour; Scale bar = 20 μm). **d** The protocol used to determine the kinetics of FM1-43 release from synaptic terminals. Neurones were labelled by exposure to 5 μM FM1-43. **e** Normalized fluorescence intensity decay of puncta to the initial FM1-43 loading intensity was plotted. Each point represents the average of a total of 20 boutons from 10 different brain slices (*n* = 5 mice). **f** The rate of puncta unloading in brain slices ($1/_{t1/2}$) was plotted and shown. 2ANOVA with Tukey' *post hoc* test was performed with $F_{(3, 27)} = 12.08$, $P < 0.0001$. The specific *P* values for comparison between groups were shown in the graph. All data were mean ± s.e.m (n = 5 mice), unless sated otherwise. Source data are provided as a Source Data file.

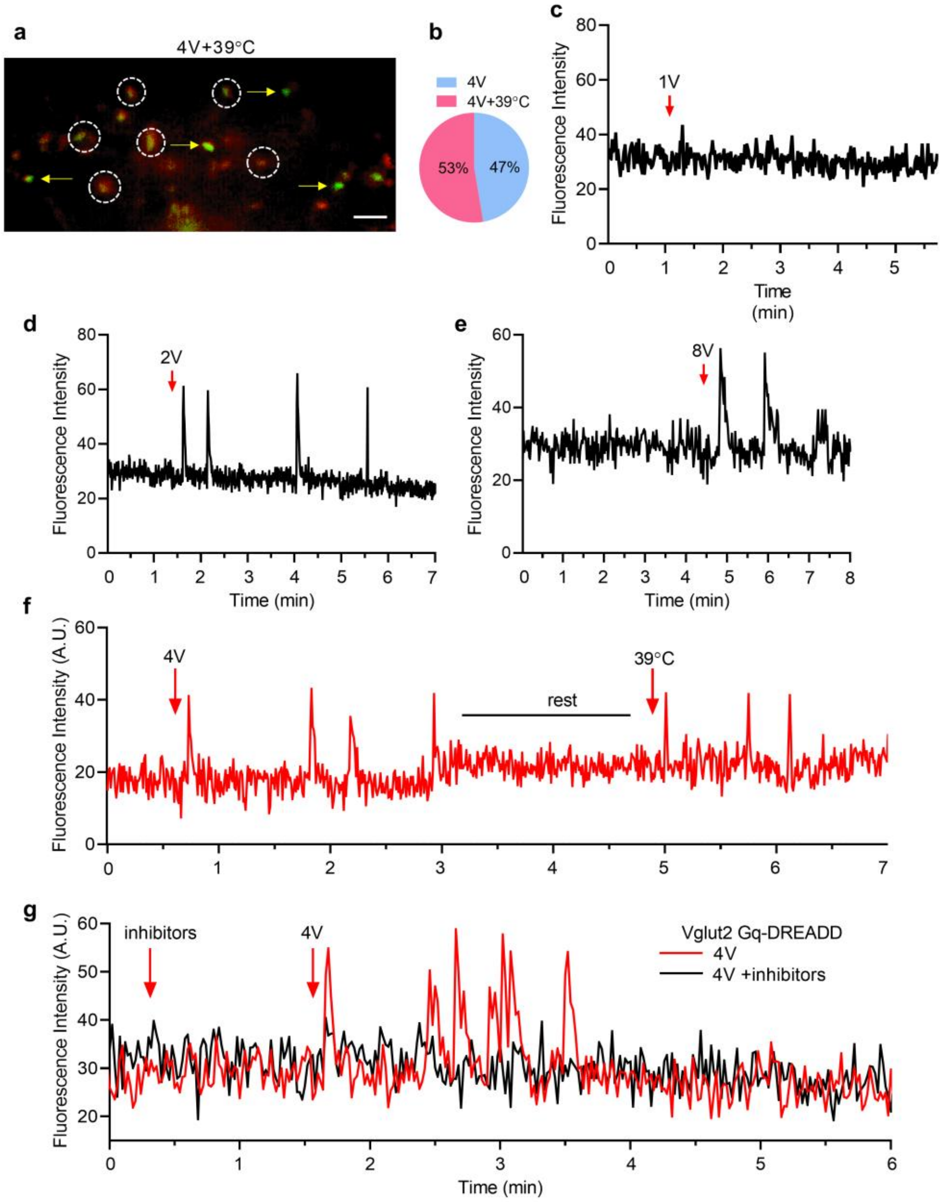

Supplementary Fig. 9 | HFS activated intracellular calcium transients in WSNs in the medial preoptic nucleus. a C57BL/6J mouse brain slices were loaded with Fluo-8 to detect calcium transients. A fluorescent image showing cells responded to 4 V HFS (green colour) and thermal stimulation at 39 °C (circled yellow-coloured cells). Yellow-coloured arrows indicated cells only responded to 4 V HFS. Scale bar = 50 µm. **b** A pie chart showing cells positively responded to both 4 V HFS and 39 °C stimulations (red colour). **c - e** Calcium transient traces from brain slices stimulated with 1 V, 2 V, and 8 V HFS. The same brain slice was allowed to rest for 4 min and then stimulated at 39 °C (**f**) to show increased calcium transient spikes. **g** Vglut2-Cre mouse was injected with Gq-DREADD-AAV-mCherry. The brain slices were loaded with Fluo-8 to detect calcium transients with 4 V HFS (red line) and with a cocktail of synaptic blockers (black line). n = 5 mice (20 cells) for calcium studies. Source data are provided as a Source Data file.

2. Supplementary Table 1

Supplementary Table 1 | Statistical results for the Supplementary Fig. 4c-e.

Fig. 4 panels	Time	1ANOVA with Tukey's post hoc test	Sham vs. tMCAO-0.5h	Sham vs. tMCAO1h	tMCAO-0.5h vs. tMCAO-1h
**c,**	1st night	F(2, 204)=5.195, P = 0.0063	*P*<0.0001(####)	*P*< 0.0001 (####)	*P*=0.3613 (ns)
	1st day	F(2, 95)=6.084, P = 0.0027	*P*<0.0001(####)	*P*< 0.0001 (####)	*P*=0.1556 (ns)
**d,**	3rd night	F(2, 159)=11.82, P< 0.0001	*P*=0.0017 (##)	*P*< 0.0001 (####),	*P*=0.1154 (ns)
	3rd day	F(2, 240)=6.165, P = 0.0024	*P*<0.0001(####)	*P*< 0.0001 (####)	*P*=0.9928 (ns)
**e,**	5th night	F(2, 198)=12.33, P< 0.0001	*P*=0.1004 (ns)	*P*= 0.1617 (ns)	*P*=0.0004 (###)
	5th day	F(2, 198)=3.155, P<0.0448	*P*=0.0050 (##)	*P*<0.0001 (####)	*P*<0.0001 (####)
