# Supplementary material for: Hypothermia evoked by stimulation of medial preoptic nucleus protects the brain in a mouse model of ischaemia
Source: Nat Commun. 2022 Nov 12;13:6890. doi: 10.1038/s41467-022-34735-2 (PMC9653397; doi:10.1038/s41467-022-34735-2)
Supplement: Supplementary file 3 — Description of Additional Supplementary Files [file 41467_2022_34735_MOESM3_ESM.pdf]

Description of Additional Supplementary Files:

Supplementary Movie 1 I Recordings of the hypothermic mice behaviours. These videos were captured of mice during the normothermic condition (Video 1) , hypothermia-evoked using chemogenetics (Video 2), surface-cooling (Video 3), and DBS (Video 4). Visible shivering and increased breathing rate can be seen in surfacecooling mice.
