# Supplementary material for: Hypothermia evoked by stimulation of medial preoptic nucleus protects the brain in a mouse model of ischaemia
Source: Nat Commun. 2022 Nov 12;13:6890. doi: 10.1038/s41467-022-34735-2 (PMC9653397; doi:10.1038/s41467-022-34735-2)
Supplement: Supplementary file 5 — Reporting Summary [file 41467_2022_34735_MOESM5_ESM.pdf]

nature portfolio

Corresponding author(s): Sheng-Tao Hou

Last updated by author(s): Oct 28, 2022

Reporting Summary

Nature Portfolio wishes to improve the reproducibility of the work that we publish. This form provides structure for consistency and transparency in reporting. For further information on Nature Portfolio policies, see our Editorial Policies and the Editorial Policy Checklist.

Statistics

For all statistical analyses, confirm that the following items are present in the figure legend, table legend, main text, or Methods section.

n/a	Confirmed	
☐	☒	The exact sample size (n) for each experimental group/condition, given as a discrete number and unit of measurement
☐	☒	A statement on whether measurements were taken from distinct samples or whether the same sample was measured repeatedly
☐	☒	The statistical test(s) used AND whether they are one- or two-sided<br>*Only common tests should be described solely by name; describe more complex techniques in the Methods section.*
☐	☒	A description of all covariates tested
☐	☒	A description of any assumptions or corrections, such as tests of normality and adjustment for multiple comparisons
☐	☒	A full description of the statistical parameters including central tendency (e.g. means) or other basic estimates (e.g. regression coefficient) AND variation (e.g. standard deviation) or associated estimates of uncertainty (e.g. confidence intervals)
☐	☒	For null hypothesis testing, the test statistic (e.g. F, t, r) with confidence intervals, effect sizes, degrees of freedom and P value noted<br>*Give P values as exact values whenever suitable.*
☒	☐	For Bayesian analysis, information on the choice of priors and Markov chain Monte Carlo settings
☒	☐	For hierarchical and complex designs, identification of the appropriate level for tests and full reporting of outcomes
☐	☒	Estimates of effect sizes (e.g. Cohen's d, Pearson's r), indicating how they were calculated

Our web collection on statistics for biologists contains articles on many of the points above.

Software and code

Policy information about availability of computer code

Data collection	Data and images were collected using OmniPlex (v1.13.0, Plexon), pClamp (V9, Molecular Devices), FV30-SW (Olympus), SciScan (v1.4, Scientifica), ZEN (V2.6, Carl Zeiss AG), Grip Strength Meter from Columbus Instruments (BAS-47200), thermometer (DC700 multichannel temperature recorder, DCUU), the Columbus Instruments Comprehensive Lab Animal Monitoring System (CLAMS); and the following software: pClamp (V9, Molecular Devices); ImageJ (v1.52k, NIH); Imaris (V9.2, Oxford Instruments); ZEN (V2.6, Carl Zeiss AG); MaxQuant (v.1.5.2.8, Max Planck Institute of Biochemistry).
Data analysis	The statistical analysis was performed using Graphpad Prism (v9.0.0, GraphPad Software).

For manuscripts utilizing custom algorithms or software that are central to the research but not yet described in published literature, software must be made available to editors and reviewers. We strongly encourage code deposition in a community repository (e.g. GitHub). See the Nature Portfolio guidelines for submitting code & software for further information.

Data

Policy information about availability of data

All manuscripts must include a data availability statement. This statement should provide the following information, where applicable:

- Accession codes, unique identifiers, or web links for publicly available datasets
- A description of any restrictions on data availability
- For clinical datasets or third party data, please ensure that the statement adheres to our policy

The data generated in this study are provided in the Supplementary Information/Source Data File. Source data are provided with this paper. The source data

March 2021

underlying Fig.1b,c,e-; Fig. 2a,b,c-f; Fig. 3d-l, n-q; Fig. 4c-f, h,i,k,m,n; Fig 5h,i,j; Fig. 6b,d,f,g,h,i; Supplementary Figs. 1b, c; Supplementary Fig. 2b, d-g, i-k; Supplementary Fig. 3c-d, e-f; Supplementary Fig. 4b-i; Supplementary Fig. 5a-i; Supplementary Fig. 7bb-h,j; Supplementary Fig. 8j,k,n,o,; Supplementary Fig. 9c-g are provided as a Source Data file. The moue brain atlas images were derived from http://labs.gaidi.ca/mouse-brain-atlas/?ml=&ap=0.2&dv=.

Human research participants

Policy information about studies involving human research participants and Sex and Gender in Research.

Reporting on sex and gender	Not applicable
Population characteristics	Not applicable
Recruitment	Not applicable
Ethics oversight	Not applicable

Note that full information on the approval of the study protocol must also be provided in the manuscript.

Field-specific reporting

Please select the one below that is the best fit for your research. If you are not sure, read the appropriate sections before making your selection.

☒ Life sciences ☐ Behavioural & social sciences ☐ Ecological, evolutionary & environmental sciences

For a reference copy of the document with all sections, see nature.com/documents/nr-reporting-summary-flat.pdf

Life sciences study design

All studies must disclose on these points even when the disclosure is negative.

Sample size	Sample sizes were selected based on the previous examples of similar work in the literature (Zheng LF et al., Nat Commun 2020, 11,3012). Adult male and female C57Bl/6 mice (3 months old) and adult Vglut2-Cre mice (3 months old) mice were used in this study. A total of 60 mice of the same age (3-month-old) and sex (male) were first randomly assigned to two groups (30 each) to receive Sham or MCAO surgery, respectively. For stroke behavioral analyses, each group had at least 7 mice to allow for statistical analyses. For electrophysiological recordings, at least 3 mice with more than 10 brain slices were used. These sample size allowed for acceptable statistical analyses to be performed while minimizing the number of animals for experiments dictated by the magnitude of experiment-to-experiment variation.
Data exclusions	Efforts were made to minimize the number of mice used. The inclusion criterion was based on the exact age and sex of the mice. The exclusion criterion was when the mouse failed to survive MCAO surgery at the end of the 14 d periods of the experimentation, or the DBS electrodes were mistargeted as determined through post-hoc tissue analysis. Data derived from all qualified animals were included in the analyses and presentation of the results.
Replication	To achieve meaningful statistical differences, a minimum of 7 mice per group were used in the DBS, MCAO experiments and in in vivo recordings and behavioural studies, as indicated in the figure legends. For tissue section staining experimentation, at least 3 mice per group were used. Where representative images are shown, at least three repeats have been performed with similar results.
Randomization	Mice used in the current study were randomly assigned to each group to maintain total randomization. After the surgery, mice in each group were randomly assigned to receive hypothermia treatment or without hypothermia as a control.
Blinding	Investigators were blinded to groups allocation during data collection and data analysis, as stated in the method section

Reporting for specific materials, systems and methods

We require information from authors about some types of materials, experimental systems and methods used in many studies. Here, indicate whether each material, system or method listed is relevant to your study. If you are not sure if a list item applies to your research, read the appropriate section before selecting a response.

Materials & experimental systems

n/a	Involved in the study
☐	☒ Antibodies
☒	☐ Eukaryotic cell lines
☒	☐ Palaeontology and archaeology
☐	☒ Animals and other organisms
☒	☐ Clinical data
☒	☐ Dual use research of concern

Methods

n/a	Involved in the study
☒	☐ ChIP-seq
☒	☐ Flow cytometry
☒	☐ MRI-based neuroimaging

Antibodies

Antibodies used

The Supplementary Source file listed all Key Reagents. Specifically, the antibodies are rabbit anti-c-Fos (Abcam, ab208942, USA, 1:500), rabbit anti-BDNF (Abcam ab108319, 1:300), Alexa Fluor 594-conjugated goat anti-mouse secondary antibody (ab150116, Abcam, USA. 1:500) , Alexa Fluor 488-conjugated goat anti-rabbit IgG (ab150077, Abcam, USA. 1:500). The dilutions of these antibodies used have been described in the Source data file under Key reagents and in the Methods section.

Validation

All antibodies are commercially available and have been extensively validated by the manufacturer as shown on their websites.

Animals and other research organisms

Policy information about studies involving animals; ARRIVE guidelines recommended for reporting animal research, and Sex and Gender in Research

Laboratory animals

Both sexes of adult male C57Bl/6 mice (3 months old), adult male Vglut2-ires-cre knock-in (C57BL/6J) mice (3 months old) mice and Adcyap1-Cre (strain #030155, B6.Cg-Adcyap1tm1.1(cre)Hze/ZakJ) mice were used in this study. C57BL/6J mice were purchased from the Vital River Laboratory Animal Technology Co., Ltd. (Zhejiang, China). Vglut2-Cre mice (strain #028863) and Adcyap1-Cre mice (strain #030155, B6.Cg-Adcyap1tm1.1(cre)Hze/ZakJ) were purchased from the Jackson Laboratory (USA).

Wild animals

No wild animals were used in the study.

Reporting on sex

The DBS and chemogenetics data apply to both sexes. However, the tMCAO experiments were performed using only male mice, as the female mice would produce inconsistent stroke infarctions which is well documented in the literature.

Field-collected samples

No field collected samples were used in the study.

Ethics oversight

All animal experiments were conducted according to protocols approved by the Animal Care Committee of the Southern University of Science and Technology (Shenzhen, China; SUSTech-JY20202005). Efforts were made to minimize the number of animals used. ARRIVE guidelines were followed.

Note that full information on the approval of the study protocol must also be provided in the manuscript.
